# Cuticle Thickening in a Pyrethroid-Resistant Strain of the Common Bed Bug, *Cimex lectularius* L. (Hemiptera: Cimicidae)

**DOI:** 10.1371/journal.pone.0153302

**Published:** 2016-04-13

**Authors:** David G. Lilly, Sharissa L. Latham, Cameron E. Webb, Stephen L. Doggett

**Affiliations:** 1 Department of Medical Entomology, The University of Sydney and Pathology West–ICPMR Westmead, Westmead Hospital, Westmead, NSW, 2415, Australia; 2 Australian Centre for Microscopy & Microanalysis, The University of Sydney, NSW, 2006, Australia; University of Crete, GREECE

## Abstract

Thickening of the integument as a mechanism of resistance to insecticides is a well recognised phenomenon in the insect world and, in recent times, has been found in insects exhibiting pyrethroid-resistance. Resistance to pyrethroid insecticides in the common bed bug, *Cimex lectularius* L., is widespread and has been frequently inferred as a reason for the pest’s resurgence. Overexpression of cuticle depositing proteins has been demonstrated in pyrethroid-resistant bed bugs although, to date, no morphological analysis of the cuticle has been undertaken in order to confirm a phenotypic link. This paper describes examination of the cuticle thickness of a highly pyrethroid-resistant field strain collected in Sydney, Australia, in response to time-to-knockdown upon forced exposure to a pyrethroid insecticide. Mean cuticle thickness was positively correlated to time-to-knockdown, with significant differences observed between bugs knocked-down at 2 hours, 4 hours, and those still unaffected at 24 hours. Further analysis also demonstrated that the 24 hours survivors possessed a statistically significantly thicker cuticle when compared to a pyrethroid-susceptible strain of *C*. *lectularius*. This study demonstrates that cuticle thickening is present within a pyrethroid-resistant strain of *C*. *lectularius* and that, even within a stable resistant strain, cuticle thickness will vary according to time-to-knockdown upon exposure to an insecticide. This response should thus be considered in future studies on the cuticle of insecticide-resistant bed bugs and, potentially, other insects.

## Introduction

Since the turn of the century there has been a dramatic resurgence across many countries in the number of infestations of bed bugs, both the common, *Cimex lectularius* L., and tropical *Cimex hemipterus* F. species [1]. Insecticide resistance has been commonly inferred as a key driver of the bed bug resurgence [1–5] as pyrethroid resistance has been found to be concomitantly widespread with the growth and spread of infestations [4, 6–16]. Several mechanisms have been discovered that contribute to the observed resistance, including *kdr*-type target-site protein mutations [8, 9, 15, 16], overexpression of detoxifying enzymes [15, 17], and reduced penetration [11, 18].

Reduced penetration in other insecticide-resistant insects has been known to be a resistance mechanism since it was first established in the 1960s with pyrethrin [19], organophosphates [20–22], carbamates [23, 24], and organochlorines [25–28]^.^ It can occur via multiple mechanisms, including enhanced expression of metabolic resistance mechanisms in the integument [18], the increased presence of binding proteins, lipids, and/or sclerotization that trap insecticides [29, 30], a measurably thicker cuticle [31, 32], or a combination of some or all of these mechanisms together [30, 33–35]. Several of these mechanisms have been found in bed bugs with, to date, the exception of cuticle thickening.

Ordinarily, reduced penetration does not, by itself, impart a high degree of resistance [25, 33, 36], although it may nonetheless have importance by way of conferring a level of cross-resistance to a wider variety of insecticides [25, 33, 37], increasing the efficiency of metabolic detoxification [26, 38–40], or delaying the onset of knockdown [25, 32, 41]. However, the expression of one or more resistance mechanisms does not necessarily predicate a corresponding change in expression of cuticular proteins [42] although it appears that, conversely, reduced penetration is typically found only when other mechanisms are present [32, 43–45].

No measurable comparison of the cuticle thickness has thus far been undertaken in bed bugs, although evidence from very recent molecular analysis of several *C*. *lectularius* strains point toward changes in protein expression [11, 39]. The manifestation and intensification of other resistance mechanisms in the cuticle is strongly indicative of reduced penetration and/or cuticle thickening [18] and this has most recently been successfully established in the South African malaria vector mosquito *Anopheles funestus* [32]. Thus, in adapting the experimental design used with *Anopheles funestus*, it is the aim of this paper to investigate the potential presence of cuticle thickening in a highly pyrethroid-resistant strain of *C*. *lectularius* and to examine the relationship of any such thickening to time-to-knockdown upon exposure to an insecticide.

## Experimental Methods

### Chemicals

Demand Insecticide^®^ (25 g/L lambda-cyhalothrin) was supplied free of charge by Syngenta Crop Protection Pty Limited (Level 1, 2–4 Lyonpark Road, Macquarie Park NSW 2113).

### Bed bug strains

Storage and culturing of the bed bug strains were conducted as approved by the Westmead Hospital Animal Ethics Committee (WHAEC Protocol No.2002) and in accordance with NSW Animal Research Review Panel (ARRP) Guidelines for the Housing of Rats in Scientific Institutions.

*Cimex lectularius* specimens were collected from a single, domestic, field infestation in the suburb of Parramatta, New South Wales, Australia, in December 2012. A detailed treatment history was not available for this strain, although it is believed insecticide control had been attempted prior to its collection. The specimens were examined for species, [46] *kdr* haplotype [8] and, thereafter, used to establish a colony in the departmental insectary maintained at 25°C (±1°C) and 75% (±10%) RH with a photoperiod of 12:12 (L:D) h [12]. All bed bugs were offered a blood meal on specific-pathogen-free (SPF) anaesthetised rats seven days prior to being selected for use in bioassays. Rat anaesthesia was achieved with an intraperitoneal injection of ketamine and xylazine, and recovery monitored until complete. No insecticide selection was undertaken. Resistance profiling indicated this strain homogeneously possessed haplotype B (L925I only) *kdr*-type resistance [8]. Exposure to a d-allethrin based rapid resistance field assay also failed to elicit any statistically significant levels of knockdown [47]. Given the lack of efficacy with d-allethrin (a first generation, type I pyrethroid), a second-generation type II pyrethroid, lambda-cyhalothrin, was preferentially selected for use as the screening agent in this study.

An additional laboratory strain of *C*. *lectularius* kept in colonies since the 1960s with known susceptibility to pyrethroids (designated the ‘Monheim’ strain [12]) was also used selectively to assess for any effect due to variations in insect body size. The Monheim strain lacks any *kdr*-type mutation (haplotype A) [8].

To ensure all bugs used in the experiments were of equal age, cohorts of fifth instar bugs were isolated from the stock colonies, fed to repletion, and monitored daily for appearance as adults. Once matured the bugs were immediately separated by sex to limit the potential for breeding, and aged for a further 8 or 9 days (depending on their intended use) without any further blood meal.

### Knockdown bioassay

A 20 mL/L solution of Demand Insecticide was mixed according to label directions and applied at the rate of 1.2 mL (to the point of run-off) to 90 mm Ø Whatman^™^ No. 1 qualitative filter papers (Cat. No. 1001–090) held in petri dishes. Sixteen replicates of 10 male Parramatta strain 9-day-old bed bugs were then immediately placed onto the treated surface (no drying period) and knockdown was recorded at 10-minute intervals up to 4 hours. Knockdown was defined as the bugs not being able to right themselves when inverted onto their dorsal side. Affected bugs were immediately transferred to labelled specimen vials containing 70% ethanol. Bed bugs that were knocked-down within the first 2 hours were marked as the ‘intolerant’ group, and unaffected bugs at 4 hours were marked as the ‘tolerant’ group. The experiment was repeated until at least 10 bugs were collected for each response group.

In order to control for the additional time required in selecting for the most resistant bed bugs, the above experiment was repeated with a cohort of 8-day-old Parramatta strain male bugs, and the experiment (9 replicates of 10 bugs) allowed to run for 24 hours of continuous forced exposure. Any bed bugs unaffected at the conclusion of 24 hours were then marked as the ‘resistant’ group, and were thus 9 days old at the point of sampling as per the ‘intolerant’ and ‘tolerant’ response groups.

Exposure of the Monheim strain bugs to the above regimen was attempted, however, all bugs rapidly succumbed to the insecticide within a single 10-minute period and thus no separation into groups of varying tolerance was possible. Consequently, the Monheim strain was excluded from the inter-group cuticle thickness and time-to-knockdown analysis, but a random sample of 9-day-old bugs were nonetheless processed for cuticle measurements in order to provide for a comparison between ‘susceptible’ and ‘resistant’ bugs.

### Preparation for scanning electron microscope

Specimens preserved in 70% ethanol were examined under the dissecting microscope in fresh 70% ethanol. The middle legs of each specimen were dissected from the body at the apical region of the femur. A subsequent severance was made at the tibia midpoint whilst the legs were suspended in a small drop of 70% ethanol. The remaining specimen bodies were stored in 70% ethanol for later analysis. Legs were moved to Eppendorf tubes, washed twice more in 70% ethanol to remove sectioning debris and processed in an ascending ethanol series as follows: 80%, 90%, 95% and 100%, twice for 5 mins each, followed by 3 washes for 10 minutes each in ultrapure ethanol. Samples were chemically dried for 3 mins in hexamethyldisilazane (Proscitech), transferred to new Eppendorf tubes and air-dried for 5 mins. SEM specimen stubs (Ted Pella) were modified with a diamond saw to expose a flattened surface. Legs were mounted to this surface with carbon tape and carefully positioned to ensure the tibia was vertically positioned and the severed midpoint was exposed at the stubs apical surface. Carbon paint was applied to secure samples to the specimen stubs. Specimens were sputter coated with gold for 2 mins at 25 mA with the EMITECH K550X and were stored in a desiccator.

### Electron microscope imaging

Secondary electron micrographs were obtained with a Zeiss EVO 50 SEM at 10 kV. Each sample was individually rotated and tilted towards the secondary electron detector, varying the working distance between 9 and 25 mm. Image focus and scale calibrations were corrected with the dynamic focus and tilt correction tools. Integrated line averaging was utilised to minimise charging artefacts during image acquisition.

### Image analysis

**Fig 1:**
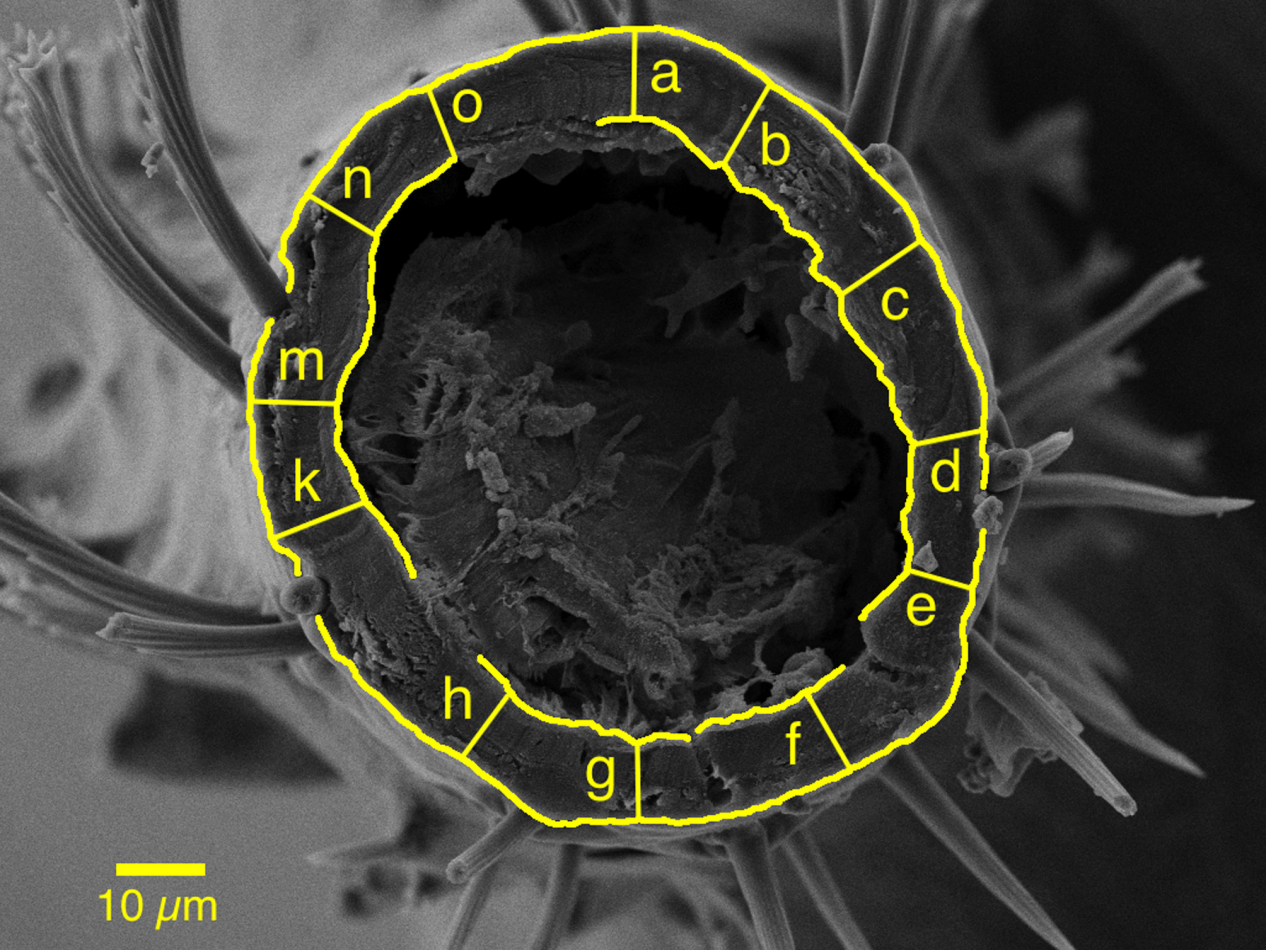
Transverse section of *Cimex lectularius* (Parramatta strain) middle leg tibia and example of twelve point-to-point cuticle measurements methodology. Letters denote measurements of: a = 9.15 μm, b = 9.82 μm, c = 10.25 μm, d = 7.91 μm, e = 7.16 μm, f = 8.42 μm, g = 8.02 μm, h = 8.41 μm, k = 10.12 μm, m = 8.82 μm, n = 8.31 μm, o = 8.09 μm [i, j and l not included for purpose of legibility].

Raw and processed images were analysed in ImageJ (version 1.46r) [48]. To assess cuticle width, the known scale in microns from the processed imaged was measured according to the number of pixels, and this value transferred to the raw image. Thereafter, 12 point-to-point measurements were made, roughly equidistantly, from the outermost to innermost visible sections of the cuticle, whilst avoiding obvious debris, damage, or setae beds (Fig 1). Measurement of the various specimens was made while blinded to the allocated response group (‘intolerant’, ‘tolerant’, ‘resistant’ and ‘susceptible’) in order to limit the potential for bias.

Sample sizes were balanced by random selection in IBM SPSS Statistic v22 for Mac (IBM Corp., 2013) with mean cuticle thickness values of the three Parramatta response groups compared via one-way ANOVA and Fisher’s Least Significant Difference (LSD) post-hoc tests. Cuticle thickness vs. time-to-knockdown was analysed by linear regression. Cuticle thickness between ‘susceptible’ and ‘resistant’ bugs was compared via a one-way ANOVA.

### Insect body measurements

Additional body measurements were made of the specimens from all four response groups used for cuticle analysis in order to check for any effect of varying body or appendage size on the resultant mean cuticle values. Several target measurements were adapted from previously published research [49], and included the pronotum width (*pw*), pronotum length (*pl*), head width (*hw*), length of the 1^st^ antennal segment (after the scape–*a2l*), hind leg tibia length (*t3l*), and hind leg tibia width (*t3w*). Measurements were made under dissecting microscope using stage and/or optical micrometers and evaluated for statistical significance and effect sizes (Partial Eta^2^ values) using general linear model multivariate analysis and Bonferroni post-hoc comparisons.

## Results

### Knockdown

**Fig 2:**
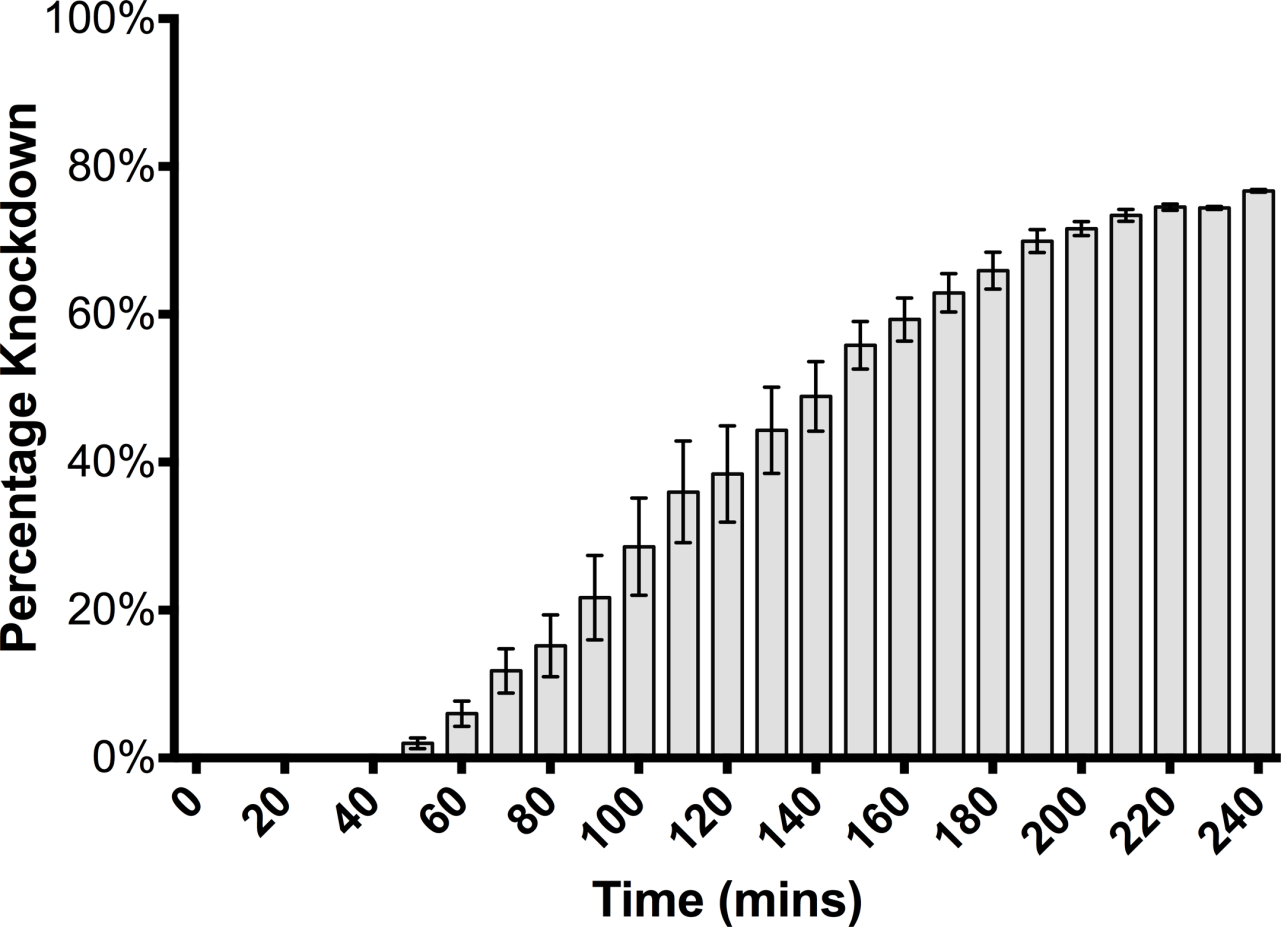
Knockdown (% ± S.E, n = 160) over time (minutes) of Parramatta strain *Cimex lectularius* upon forced exposure to wet residues of 20 mL/L Demand Insecticide^®^ (25 g/L lambda-cyhalothrin).

Knockdown of the exposed 9-day-old Parramatta-strain bed bugs occurred asymptotically up to a value of approximately 76% (S.E. ± 1.8%) over the course of 4 hours, with the first bugs affected after 50 minutes (Fig 2) and increasing up to 38% (S.E. ± 6.5%) after 2 hours (the arbitrary end-point of ‘intolerant’ response group). Bed bugs aged to 8 days and continually force-exposed for 24 hours (to an equivalent age of 9-days-old) had knockdown of 82% (S.E. ± 2.0%), leaving a further 18% of bugs that were unaffected and thereafter classed as the ‘resistant’ group.

### Imaging and cuticle thickness

Several specimens had to be excluded from analysis at the imaging stage due primarily to damage that had occurred during sectioning and mounting, or as a result of internal tissues obscuring the cuticle. Where this affected overall parity of sample size, excess specimens were excluded via random sampling in SPSS such that, in total, measurements from 10 specimens were eventually analysed from each response group.

**Fig 3:**
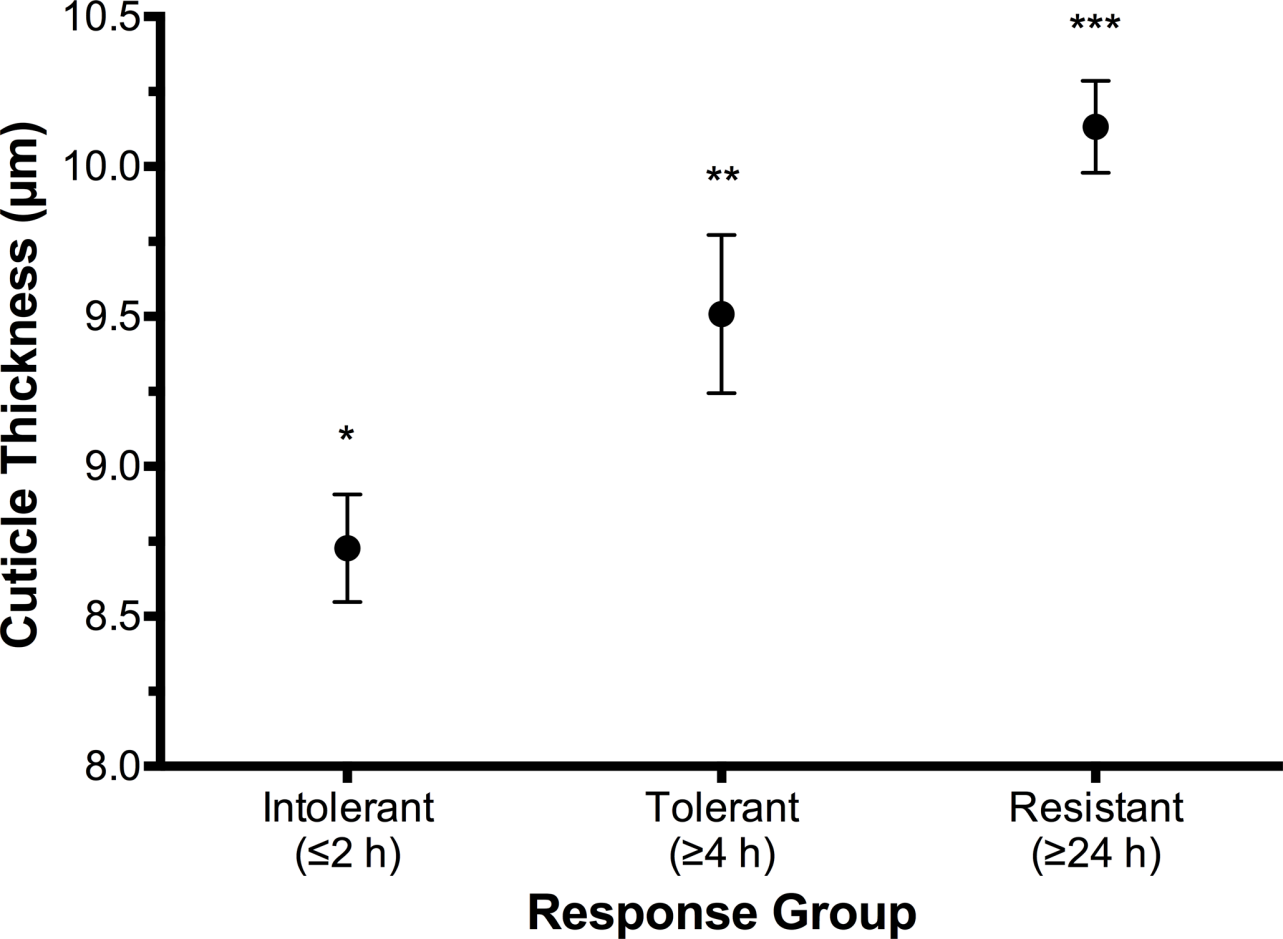
Mean cuticle thickness (μm ± S.E) of intolerant (n = 10), tolerant (n = 10) and resistant (n = 10) response group Parramatta strain *Cimex lectularius*. Asterisks (*) indicate statistical significance (p < 0.05).

**Fig 4:**
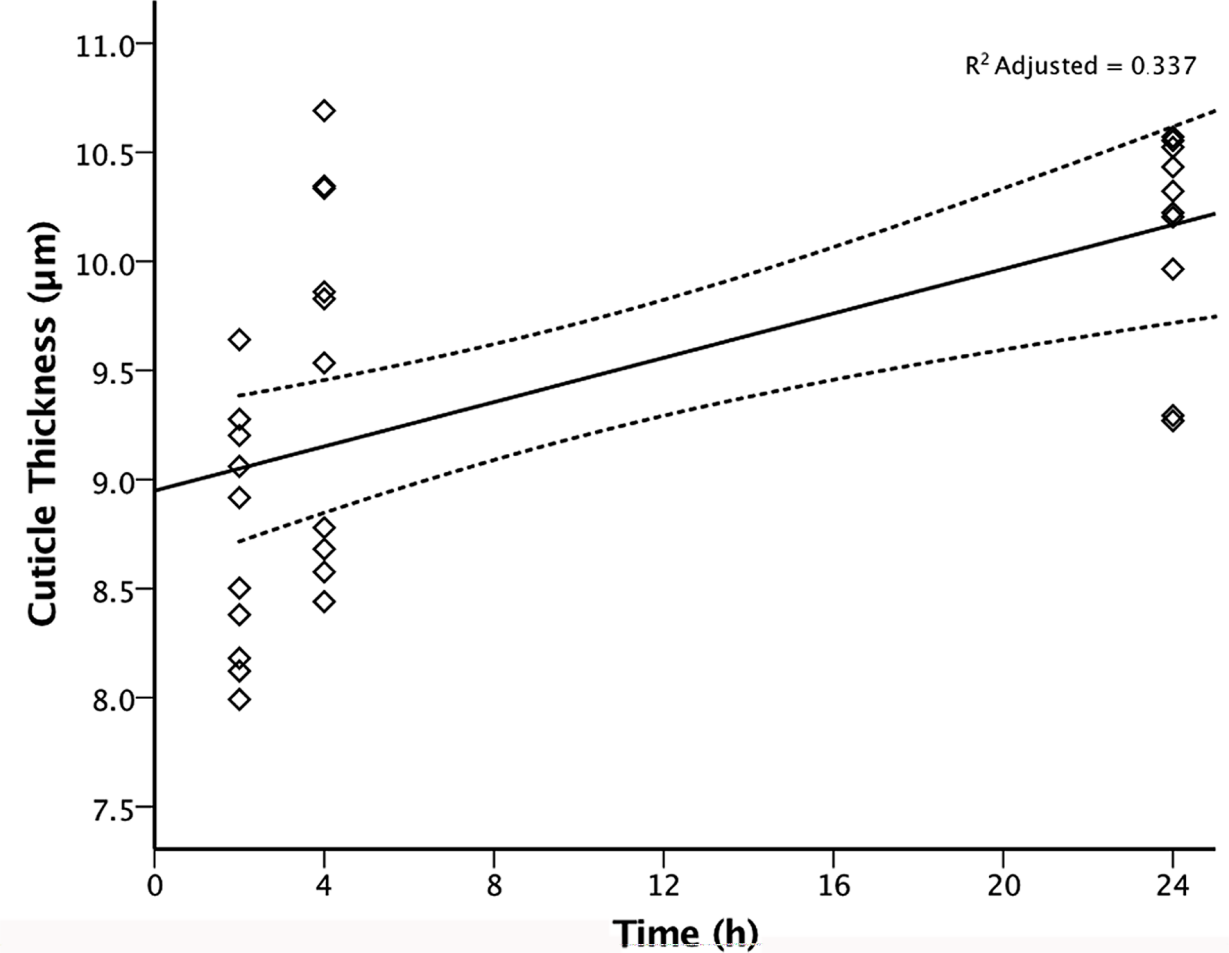
Positive correlation of time-to-knockdown to mean cuticle thickness of Parramatta strain *Cimex lectularius* (n = 10 per response group) upon continuous forced-exposure to wet residues of 20 mL/L Demand Insecticide^®^ (25 g/L lambda-cyhalothrin).

Mean cuticle thickness was found to be significantly different (p < 0.001) between the three Parramatta-strain response groups (Fig 3). ‘Resistant’ bed bugs had a mean cuticle thickness of 10.13 μm (S.E. ± 0.15 μm), ‘tolerant’ bugs 9.51 μm (S.E. ± 0.26 μm) and ‘intolerant’ bugs 8.73 μm (S.E. ± 0.18 μm). ‘Resistant’ bugs thus had a significantly different mean cuticle thickness of +0.62 μm and +1.4 μm compared to the ‘tolerant’ (p < 0.38) and ‘intolerant’ (p < 0.001) bugs respectively, and the ‘tolerant’ bugs were also found to have a significantly different mean cuticle thickness of +0.78 μm compared to the ‘intolerant’ bugs (p < 0.012). Linear regression analysis of the time-to-knockdown and mean cuticle thickness (Fig 4) revealed a significant trend (p < 0.01), with cuticle thickness positively correlated (R^2^ adjusted = 0.337) with increasing time-to-knockdown. Given the relatively limited sample sizes and length between time intervals the otherwise modest adjusted R^2^ value is, in context, a valid result.

### Insect measurements

No statistically significant differences existed within the Parramatta strain between the three response groups for all the various body measurements (pronotum width, pronotum length, head width, length of the 1^st^ antennal segment, hind leg tibia length, hind leg tibia width and ratio of tibia width to cuticle thickness), except cuticle thickness as noted separately above.

Monheim strain pyrethroid-susceptible bugs were found to be statistically significantly larger than the Parramatta strain bugs in pronotum width (all groups p < 0.001), pronotum length (‘intolerant’ p < 0.002; ‘tolerant’ p = 0.042), head width (all groups p < 0.001), antennal length (all groups p < 0.002), and tibia length (all groups p < 0.002). No statistical significant differences were observed for tibia width (all groups p > 0.05), and pronotum length (‘resistant’ p > 0.05).

**Fig 5:**
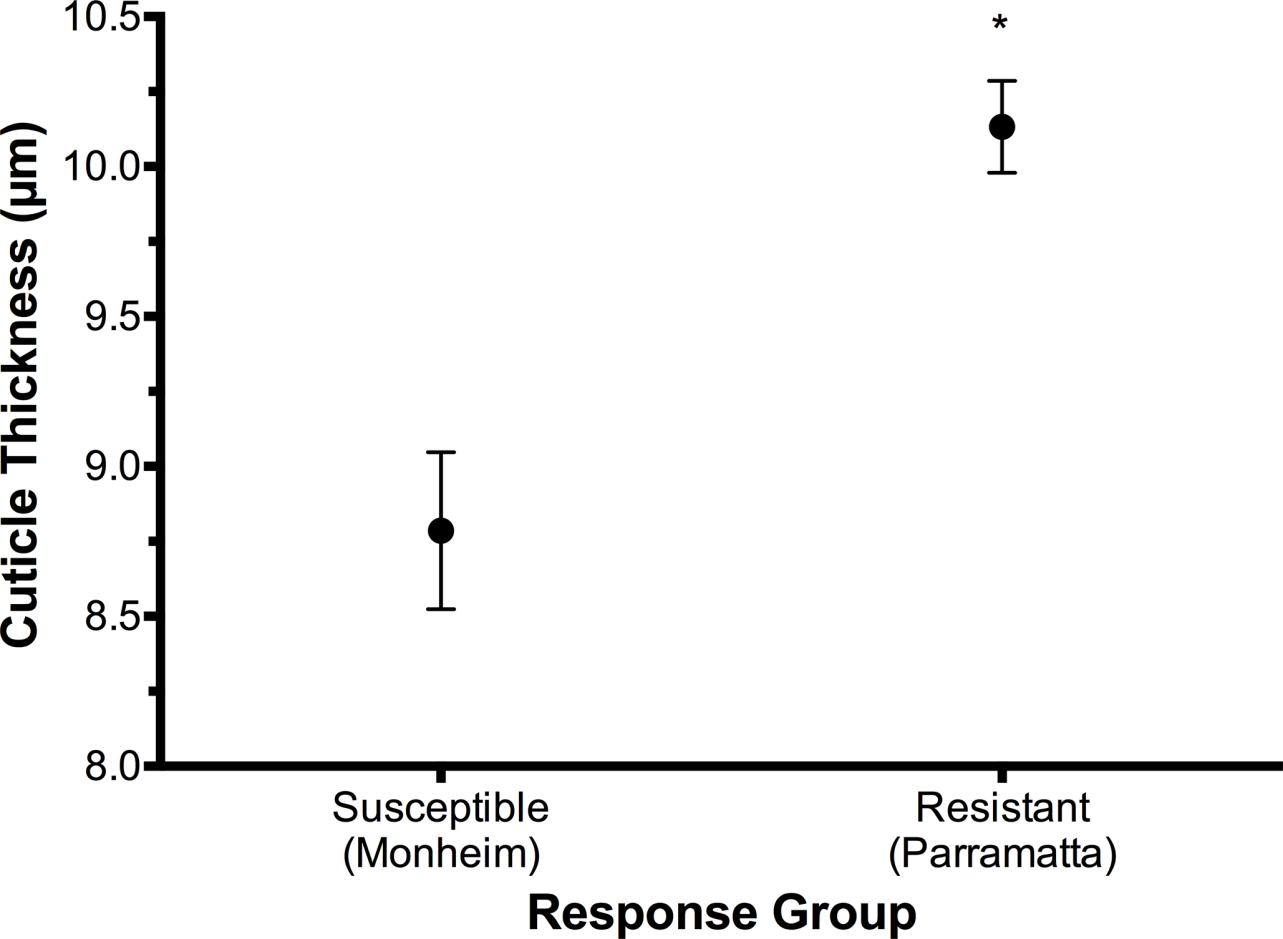
Mean cuticle thickness (μm ± S.E) of Monheim susceptible (n = 10) and Parramatta resistant (n = 10) *Cimex lectularius*. Asterisks (*) indicate statistical significance (p < 0.05).

Previously, head width in *C*. *lectularius* has been established as correlated both to differences in the size of specimens between strains and differences in body extremities relative to overall body size [49]. In this study, examination of Partial Eta^2^ (corrected) values similarly indicated that both pronotum width (0.730) and head width (0.759) were also prominent factors in accounting for the proportion of variance associated with each of the aspects measured. Consequently, head width was selected as the most appropriate feature to correct for size differences between the Monheim and Parramatta bed bug strains in order to make an appropriate comparison of mean cuticle thickness. To this effect, the observed difference in mean head width between the Monheim strain and Parramatta ‘resistant’ group bugs was found to be +9.9%. When this strain size difference is adjusted and factored into the cuticle thickness comparison, ‘resistant’ bugs had a significantly different (p < 0.001) mean cuticle thickness of +1.35 μm compared to the ‘susceptible’ bugs (Fig 5).

## Discussion

Insecticide resistance is a genetic change in response to selection by toxicants that impairs control in the field [50] and is considered to be a natural evolutionary response to human-induced environmental stress [33, 51–53]. In the case of bed bugs, this would be failure of pest management attempts at infestation eradication through insecticidal means. Development or enhancement of the cuticle as the primary barrier to insecticides is integral to this process with an obvious advantage (in the absence of any other fitness costs) conferred to the bed bug phenotype of higher tolerance to forced insecticide exposure. Using a well-established time-to-knockdown method enables identification and separation of bugs of varying insecticide tolerance. In this study, the most resistant bed bugs (themselves separated from a highly resistant field strain) were found to have cuticle thickness 16% greater than the least resistant (‘intolerant’) bugs and 6.5% from the intermediately resistant (‘tolerant’) bugs. Further analysis (after correcting for strain size differences) indicated the ‘resistant’ bugs possessed cuticle thickness 15.3% greater than that of a susceptible strain, thereby confirming a link between increased cuticle thickness and observed insecticide resistance.

Under normal field circumstance it would be expected that some variation in cuticle phenotype within a strain could be apportioned to factors such as diet or age. However, both diet and age were controlled prior to forced-selection of the bugs based on research that has shown bed bugs are appreciably resistant and express a stable metabolism at 9 days [54, 55], and that no difference in efficacy has been observed regardless of whether bugs have been fed previously (e.g. at day 1) or starved for up to 9 days [56]. This aligns with the practices of other laboratories employing bed bugs in insecticide-based bioassays, with most selectively using male bed bugs, aging between 7–10 days after emergence, and not feeding for 5–8 days prior to the experiment(s), if at all [6, 11, 13, 57–59].

A difference in cuticle thickness between strains of varying insecticide susceptibility was established in this study, after corrections were made for the increased size of the susceptible strain over the resistant strain. Given the Monheim strain bed bugs were found to be larger than the Parramatta strain bugs across a range of physical features (pronotum width, pronotum length, head width, antennal length, and tibia length) it is evident that overall insect size is a factor that should be considered in any future studies targeting morphological features if more than one field strain is being examined. Use of morphological features, and particularly measurement of head width, has been used to analyse evolutionary aspects of bed bugs [49] and our results suggest a similar approach may be warranted when examining other physical mechanisms that may confer reduced insecticide susceptibility in *C*. *lectularius*.

Thickening of the cuticle in resistant bed bugs may have important consequences for field control as the delivery of insecticides becomes potentially less efficient. However, further study of the cuticle layers, the relative contribution of each to the overall thickness, and the process of cuticle development and deposition in resistant bed bugs may be required in order to understand to what degree the cuticle thickening is reducing penetration (either alone or by enhancement with other resistance mechanisms). This, in turn, may influence advancements in insecticides and formulations in an effort to overcome the increased tolerance of bed bugs. Ideally, this research should be repeated with more field strains of both the common and tropical species of bed bug, however, the high cost of SEM research (currently USD$5000 per strain) was a limiting factor in this study. Nonetheless, further work is ongoing on a full examination of other resistance mechanisms within this and other field strains from Australia.

## Conclusions

This study demonstrates that the most highly resistant bed bugs from a pyrethroid-resistant field strain possess a thicker cuticle that enables those bugs to survive exposure to insecticides when compared both to less tolerant insects from the same strain and also those from a susceptible strain. This may help to explain why failures in the control of field infestations are so common, and further emphasizes the need for an integrated approach in the control of bed bugs to prevent the further spread of highly resistant insects. Changes in cuticle thickness, the lipid composition and passage of insecticides, if combined with expression of detoxifying enzymes or target-site insensitivity, may also have important consequences for the use of formulated insecticides for the field control of bed bugs.
